# Variations in 25-Hydroxyvitamin D in Countries from the Middle East and Europe: The Roles of UVB Exposure and Diet

**DOI:** 10.3390/nu11092065

**Published:** 2019-09-03

**Authors:** William B. Grant, Hana M. A. Fakhoury, Spyridon N. Karras, Fatme Al Anouti, Harjit P. Bhattoa

**Affiliations:** 1 Director, Sunlight, Nutrition, and Health Research Center, P.O. Box 641603, San Francisco, CA 94164-1603, USA; 2 Department of Biochemistry and Molecular Medicine, College of Medicine, Alfaisal University, P.O. Box 50927, Riyadh 11533, Saudi Arabia; 3 Division of Endocrinology and Metabolism and Diabetes Center, First Department of Internal Medicine, Medical School, Aristotle University of Thessaloniki, AHEPA University Hospital, 55535 Thessaloniki, Greece; 4 College of Natural and Health Sciences, Department of Public Health and Nutrition, Zayed University, P.O. Box 4783, Abu Dhabi 144534, UAE; 5 Endocrinology Unit and Andrology and Cryopreservation Unit, Department of Laboratory Medicine, Faculty of Medicine University of Debrecen Nagyerdei krt. 98, H-4032 Debrecen, Hungary

**Keywords:** animal fat, diet, eggs, Europe, ocean fish, latitude, Middle East, solar UVB, vitamin D deficiency, 25-hydroxyvitamin D

## Abstract

Serum 25-hydroxyvitamin D (25(OH)D) has been largely associated with latitude and sunshine exposure across several regions. According to previous results, 25(OH)D concentrations are, on average, relatively low in countries with abundant sunshine, including those of the Middle East and North Africa region, as well as lower-latitude Europe. The standard explanation for this phenomenon is that people wear concealing clothing because of cultural and religious practices and that high temperatures in summer limit direct sun exposure. However, the role of diet in the development of profound hypovitaminosis D has not been adequately explored in those countries. To examine how diet affects vitamin D status in the Middle Eastern and European countries, a search was conducted for papers from that region reporting 25(OH)D concentrations. Papers were sought that reported summertime and wintertime 25(OH)D concentrations for healthy nonpregnant adults representative of the entire population. Data from 15 Middle Eastern and European countries were found through this search. Data for postmenopausal women from 19 European countries were also obtained. Dietary supply data for animal products containing vitamin D (animal fat, eggs, ocean fish, animal meat, and milk) were obtained from the Food and Agriculture Organization of the United Nations. Latitude and a solar UVB dose index also were obtained for each country. For the 15-country study, energy from dietary factors was highly correlated with latitude, making it difficult to separate the effects of UVB exposure and dietary factors. However, for the 19-country study, dietary factors were only weakly correlated with latitude. In that study, ocean fish was the most important single dietary factor affecting serum 25(OH)D concentration for postmenopausal women in various European countries, but animal fat and meat also contributed. Because this is an ecological study, further research is encouraged to evaluate and extend the findings.

## 1. Introduction

In countries in the Middle East and North Africa (MENA) region and Southern Europe, serum 25-hydroxyvitamin D (25(OH)D) concentrations are generally lower than those in Europe and the United States [1]. According to a recent review of 41 observational studies from the MENA region, hypovitaminosis D—defined as a serum level of 25(OH)D < 50 nmol/L—ranged from 11% to 96% in children and adolescents and from 54% to 90% in adults [2]. Moreover, a recent analysis reported that more than 30% of the population in Southern Europe is vitamin D deficient (<20 ng/mL, or <50 mmol/L) [3].

In the MENA region, one reason for low 25(OH)D concentrations is the cultural and religious practice of covering the body with concealing clothing [4]. That practice is relevant to countries where most of the population practices Islam. Veiled women have lower 25(OH)D concentrations than those of unveiled women in Tunisia [5], Jordan [6], and Lebanon [7]. However, a study of 25(OH)D concentrations showed little difference in women with different amounts of skin area exposed to sunlight [8]. A second reason for low 25(OH)D concentrations is the hot summers in Arabian Gulf countries, encouraging people to stay in air-conditioned buildings in summer. Therefore, 25(OH)D concentrations are lower in summer than in winter in Bahrain [9] and Saudi Arabia [10].

Another factor is that vitamin D fortification of food in the MENA region is limited [11]. Most inhabitants do not take vitamin D supplements, whereas many people in Iran do [12]. In addition, although vitamin D food fortification strategies were implemented in Northern Europe decades ago, Mediterranean countries with abundant sunshine, including Greece, Italy, and Spain, have not yet adopted similar policies [13].

A factor generally overlooked, especially in the MENA region, is that animal products are an important source of vitamin D. A study from Lebanon in 2005 regarding dietary vitamin D intake acknowledged that animal products contained vitamin D (100 ± 70 IU/d in the Lebanese diet), but overlooked the fact that animal products also can contain 25(OH)D [14]. In a UK study on diet and serum 25(OH)D for predominantly white European participants, researchers found that meat eaters had the highest 25(OH)D concentrations throughout the year [15]. The adjusted geometric means were as follows: meat eaters, 77 nmol/L; fish eaters, 72 nmol/L; vegetarians, 66 nmol/L; and vegans, 56 nmol/L. The reason for that finding is that most animal products other than milk also have vitamin D as 25(OH)D [16,17,18].

This paper evaluates the hypothesis that dietary animal products are an important source of vitamin D and that low–animal product diets in the MENA region are an important reason for low 25(OH)D concentrations. In addition, the study explores the relationship between latitude, a possible index for UVB exposure, and 25(OH)D concentration in the Middle East and Europe.

## 2. Methods and Materials

To evaluate the diet–25(OH)D hypothesis, we obtained dietary supply values and serum 25(OH)D concentrations from countries in the Middle East and European regions. Dietary supply values (in kilocalories/person/day) for animal fat, eggs, ocean fish, meat (bovine, mutton, pig meat, poultry, other, and offals), and milk were obtained from the Food and Agriculture Organization (FAO) data for the year when the 25(OH)D data were obtained [19]. However, if the 25(OH)D data were obtained after 2013, data for 2013 were used because that is the last year for which data are available. Those values represent the supply of food available to the general population. In general, an estimated 70% of the supply is actually consumed, with 30% lost to spoilage or waste [20]. The FAO dietary supply data have been used in several studies examining the role of diet in the risk of disease, starting with the paper by Armstrong and Doll regarding dietary links to various types of cancer [21].

Serum 25(OH)D concentration data were obtained largely through searching the National Library of Medicine’s pubmed.gov database for the terms “25-hydroxyvitamin D” and “vitamin D”, as well as country names. In addition, some studies were found in review papers. The criteria for inclusion in the present study were that subjects were nonpregnant adults and that subjects were representative of the healthy adult population. An additional criterion was that separate values were given for summer and for winter, because it was assumed that solar UVB exposure would be the most important source of vitamin D in summer, whereas diet would be an important source in winter.

In addition, data on 25(OH)D concentration for postmenopausal women in 19 European countries were obtained from a paper reporting findings from a clinical trial on bazedoxifene for treating osteoporosis [22]. The women were enrolled between December 2001 and September 2003. The 25(OH)D concentrations were measured at the Covance Central Laboratory by the DiaSorin 25(OH)D assay with an intra-assay coefficient of variation between 8.2% and 11%. It is assumed that those study participants were generally healthy and representative of the elderly population in each country. The authors noted that 25(OH)D concentrations had significant direct correlations with both latitude and per capita gross domestic product.

No adjustment was made for the 25(OH)D assay used, although during the early period of this study, results varied considerably depending on the assay used [23,24]. However, 25(OH)D concentrations measured using BioSource assays were omitted because that assay reports much higher values than other assays.

UVB dose data were obtained from a table in a paper estimating the effect of UVB dose on pancreatic cancer incidence by country [25]. The values were based on those that NASA derived for solar radiation at the top of the atmosphere for the population center for each country at solar noon on the date of the winter solstice, multiplied by 0.004 to account for the UVB fraction of total solar radiation and then corrected for cloud cover. No correction was made for aerosol or ozone loading or for surface elevation. Thus, it appears that these data are essentially a function of latitude with a correction for mean cloud cover.

Linear regression analyses were performed using SigmaStat 4.0 (Systat Software, San Jose, CA, USA).

## 3. Results

Overall, the searches and criteria used yielded 15 countries with useful data, nine from the Middle East and six from Europe (Table 1). For the data from Israel, Italy, and Qatar, data for multiple groups were averaged by apportioning the 25(OH)D concentration for each group by the number of participants in that group. The dietary supply values (energy/capita/day) for that data set are given in Table 2.

Data for dietary factors for postmenopausal women in 19 European countries were obtained from a paper reporting data from a clinical trial on bazedoxifene for treating osteoporosis [22] and are presented in Table 3. The data for latitude, number of participants, and serum 25(OH)D concentrations are not given here due to copyright restrictions, but are freely available in Table 1 in [22]. If there were fewer than five, the concentration was not used. For those values included in Table 5, the number of participants ranged from five to 456, with the median number ~45 in both winter and in summer. That data set avoids three problems associated with the data set in Table 1—that is, the participants were similar, the 25(OH)D assay was identical, and vitamin D supplement use was very low.

The linear regression analysis results for 25(OH)D concentration as a function of latitude, UVB dose, and dietary factors for the set of 15 countries (12 of which have dietary supply data) in the Middle East and Europe are given in Table 4, whereas the results for the 19 European countries for postmenopausal women are given in Table 5. The cross-correlation coefficients for UVB dose and various dietary factors are given for these two sets of data in Table 6; Table 7. For the 15-country study, the dietary supply factors are highly correlated with latitude while in the 19-country study they are not. Thus, for the 15-country study, it is difficult to separate the effects of UVB dose from the effects of diet. The regression results for the 19-country study suggest that ocean fish is the most important single dietary factor affecting serum 25(OH)D concentration for postmenopausal women in various European countries, but that animal fat and meat also contribute.

**Figure 1:**
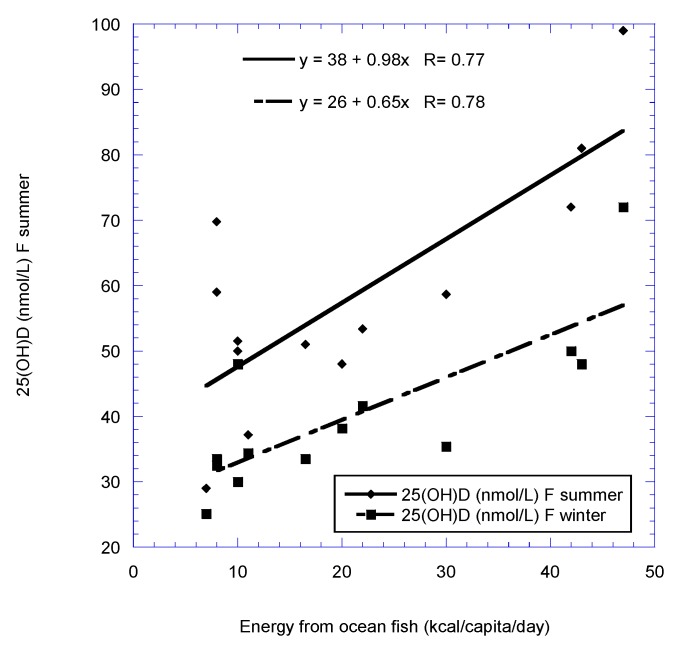
Serum 25(OH)D concentrations for females in 11 countries vs. energy supply from ocean fish (Portugal is omitted) (Table 2).

**Figure 2:**
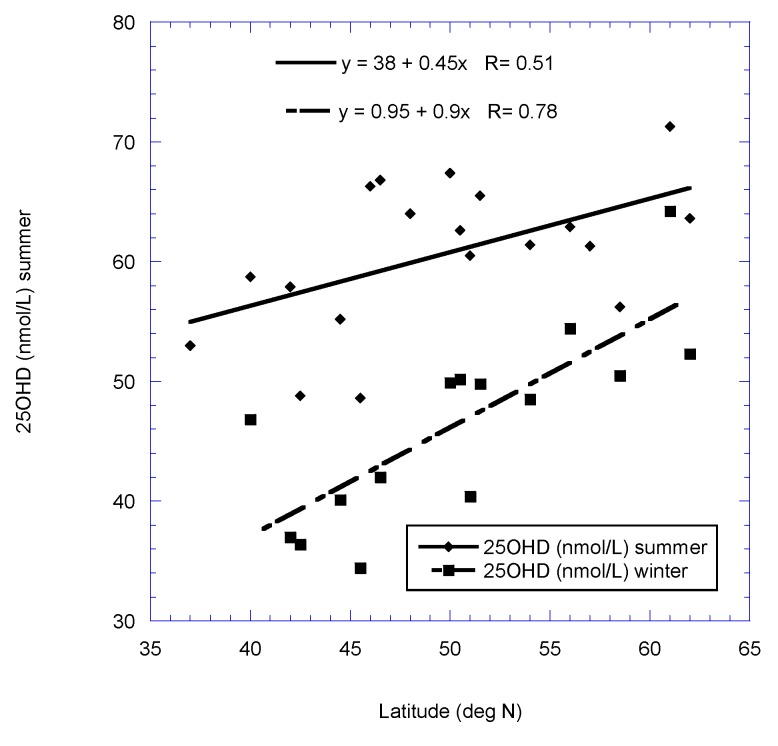
Plot of 25(OH)D concentration vs. latitude for the 19-country European postmenopausal women’s study [22].

**Figure 3:**
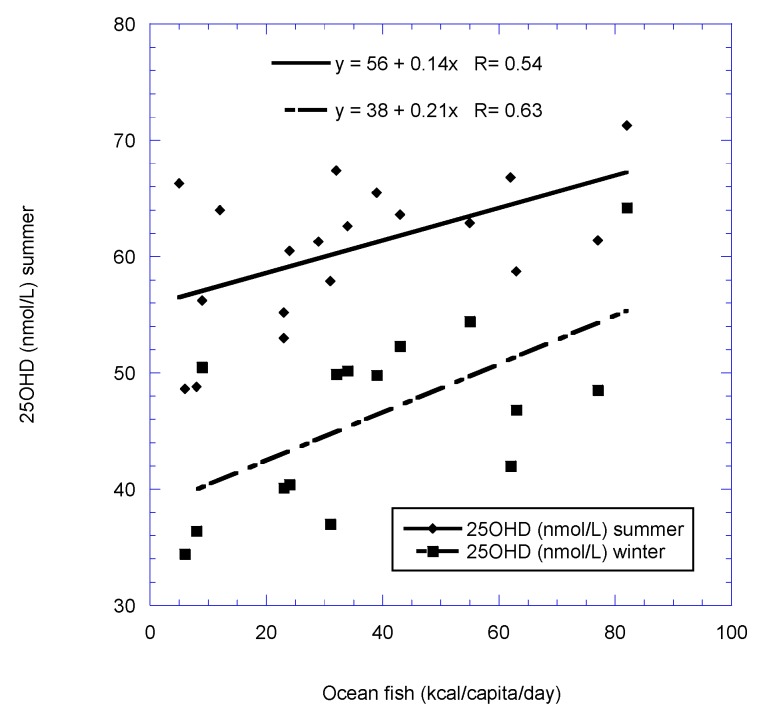
Plot of 25(OH)D concentration vs. dietary supply of ocean fish for the European postmenopausal women’s study [22].

**Figure 4:**
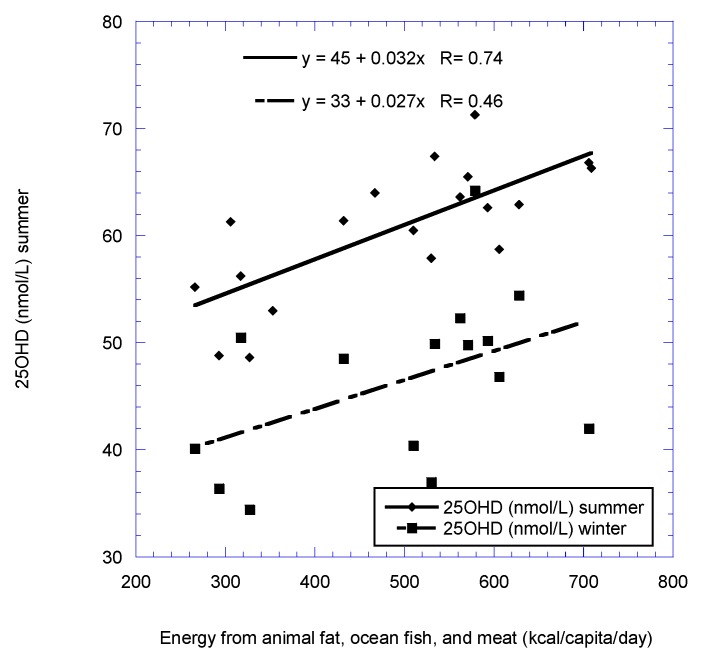
Serum 25(OH)D concentration vs. energy supply from animal fat, ocean fish, and meat for the European postmenopausal women’s study [22].

Figure 1, Figure 2, Figure 3 and Figure 4 show scatter plots of 25(OH)D concentration vs. latitude for the two country data sets, as well as for dietary supply of ocean fish for the European postmenopausal women’s study.

## 4. Discussion

The results of these ecological studies indicate that dietary factors may play a role in the higher 25(OH)D concentrations at higher European latitudes. The higher 25(OH)D concentrations in summer than in winter could be due to the combined effect of human and animal solar UVB exposure, resulting in both higher endogenous vitamin D production and oral vitamin D intake.

The finding regarding latitude is difficult to interpret. As shown in a pair of papers [41,42], skin pigmentation for indigenous humans in the Middle East and Europe lightens in relation to prevailing UV doses. That relationship is considered an evolutionary adaptation that provides photoprotection against free radical production and folate destruction at a level that also permits adequate production of vitamin D. In addition, oily fish has been a source of vitamin D in winter in Europe [43]. Thus, in general, latitude might have a limited effect on serum 25(OH)D concentrations related to UVB production from solar UVB. However, several factors might help explain that finding. One is that in predominantly Muslim countries, women tend to wear concealing clothing. Table 8 presents results regarding the effect of women being veiled or not veiled. Modest to large differences in 25(OH)D concentration can result depending on the type of clothing worn. However, although men are not veiled, they may wear robes that cover much of the body. A second factor is that it can be very hot in summer at lower latitudes. Thus, inhabitants of lower-latitude countries may elect to stay indoors where it is air conditioned. A third factor is that skin pigmentation becomes paler with increasing latitude to facilitate the production of vitamin D [44]. A fourth factor is that days are longer at higher latitudes in summer, giving inhabitants at higher latitudes more time to obtain UVB.

Use of the FAO dietary supply factors has strong support based on a review of key dietary ecological studies and their impact. The first major ecological study to examine the link between dietary factors and cancer incidence was by Armstrong and Doll [21]. As of 1 August 2019, that report had 2882 citations according to scholar.google.com. That study was extended and its results generally confirmed in a paper that used multiple linear regression analysis [48]. That paper inspired conducting the first study to identify the macrodietary factors, including fish, related to Alzheimer’s disease (AD) [49]. That ecological study resulted in researchers at Columbia University embarking on a lengthy investigation of the role of diet in the risk of AD [50,51,52]. The finding regarding fish was confirmed in 2003 [53]. In the most recent ecological study of diet and AD, meat was the most important factor, and the Mediterranean diet reduced the risk of AD by about half [54].

The results of an ecological study which found that sweeteners were an important risk factor for acute myocardial infarction for women [55] were rejected by the American Heart Association at the time because the association was unaware that triglycerides from sugars could clog the arteries through the production of triglycerides. The association accepted that mechanism in 2009 [56].

One important implication of the present study is that it casts considerable doubt on the assumption in multicountry studies that serum 25(OH)D concentrations are directly correlated with solar UVB doses, or its proxy, latitude, as is done for cancer [25]. However, that assumption seems correct in single-country studies (e.g., for cancer [57]). Thus, multicountry studies related to serum 25(OH)D concentrations should consider using 25(OH)D concentration data from each country, perhaps in conjunction with the UVB data, but should also consider skin pigmentation and clothing styles.

Several studies have investigated the amount of 25(OH)D in animal products, which is important for two reasons: it can be more important in raising human 25(OH)D concentrations than the vitamin D_3_ in the food; and because it is not included in most food frequency tables, it is generally not included in human studies involving dietary sources of vitamin D. A review from 2013 tabulated findings from various countries for vitamin D and 25(OH)D in raw meat and offal, milk and dairy products, chicken eggs, and fish [18], and found that meat and egg yolk had the highest values. However, the present study did not confirm the finding regarding meat and high 25(OH)D concentrations. A U.S. study measured the vitamin D and 25(OH)D content of several meat products, as well as eggs [17]. Beef, chicken, and pork meat had 0.9–1.4 μg/100 g of vitamin D + 25(OH)D (VitDE); beef fat and poultry skin had 2.2–2.75 μg/100 g of VitDE; and egg had 5.75 μg/100 g of VitDE. The researchers estimated that men consumed 4.15 μg/d of VitDE from meat and eggs, whereas women consumed 2.48 μg/d.

Another study measured the VitDE in retail white fish and eggs in Australia [18]. The VitDE in cooked white fish ranged from 2.2 to 3.0 μg/100 g, whereas that for hard-boiled eggs ranged from 2.4 to 6.5 μg/100 g, averaging ~4.2 μg/100 g. Those researchers also tabulated results from the Netherlands, the UK, and the U.S., finding lower values for those countries. The lower values could be due to a combination of lower solar UVB doses, increased consumption of farmed versus wild salmon, and, for eggs, keeping laying hens indoors [58].

A study from Taiwan, including 5664 community-dwelling participants aged 55 years or above, starting in 2008, examined the link between dietary factors and serum 25(OH)D concentrations [59]. Fish consumption was correlated with 25(OH)D concentrations to the *p* < 0.001 level, with >68.6 g/d intake vs. <12.9 g/d associated with a 14-nmol/L increase for males, whereas for females, >68.6 g/d vs. 0 of rarely was associated with an 11-nmol/L increase in 25(OH)D. Milk consumption was associated with much smaller increases in 25(OH)D concentrations, whereas egg and meat consumption were nonsignificantly inversely correlated with 25(OH)D concentration.

The UK study showed that fish eaters had 8-nmol/L higher 25(OH)D concentration than vegetarians and 18 nmol/L higher than vegans [15]. A meta-analysis of increases in 25(OH)D concentration from eating fatty fish indicated an increase of 7 nmol/L (95% confidence interval, 4 to 10 nmol/L) [60]. Thus, the increase in 25(OH)D for high vs. low fish supply is slightly higher than expected, which might be due to contributions from other animal products associated with fish or human solar UVB exposure.

The findings of the present study indicate that apart from the well-demonstrated effects of clothing habits, lack of food fortification policies, and sun exposure, the effect of specific dietary patterns followed in these regions might also constitute a key factor for the prevalence of hypovitaminosis D, including in populations such as the elderly, children, and pregnant women.

Although commercial 25(OH)D assays have come a long way, the results obtained by different methodologies are still not fully comparable. Now, all assays dedicated to measure 25(OH)D measure total 25(OH)D, that is, D_2_ + D_3_. Nonetheless, it has been repeatedly proven that different methodologies result in different levels of bias, either positive or negative [61,62,63,64]. It can be well recognized from summary reports of the Vitamin D External Quality Assurance Scheme (DEQAS) that different assays yield different results, and no association seems to exist between the range of measurement and the extent or the direction of the bias. However, it can be stated that the differences between methodologies have been reduced tremendously in the past decade (www.deqas.org). With more than 1000 international participants, and more than 25% using the DiaSorin chemiluminescence immunoassay LIAISON platform, perhaps DEQAS reports are well suited to follow the evolution in methodology. Liquid chromatography–tandem mass spectrometry is the “gold standard” to measure total 25(OH)D, but it is available mainly in research institutes, and the growing demand in 25(OH)D measurements favors high-throughput automated approaches. Although the National Institute of Health’s Office of Dietary Supplements established the Vitamin D Standardization Program, it has laid down its guidelines to standardize historic vitamin D data and only a few studies have been done [65,66,67]. The present study is a review of data available from the regions of interest, and these historic data have not been standardized. As is generally true for all inferences drawn from nonstandardized methodologies, they contribute to the limitations of the utility of the values generated. Standardization of vitamin D is one of the terms of reference of the International Federation of Clinical Chemistry and Laboratory Medicine’s Scientific Committee on Bone Metabolism. Among other projects, the committee also propose services to reassess the true value of 25(OH)D obtained in former epidemiological or interventional studies that had used nonstandardized methods (http://www.ifcc.org/ifcc-scientific-division/sd-committees/c-bm/). Standardization of vitamin D values measured by different assays remains a challenge.

Although 25(OH)D concentrations above 50 nmol/L may be appropriate for bone health, concentrations above 75–100 nmol/L are required for many nonskeletal effects [68]. Through various approaches, researchers have linked higher 25(OH)D concentrations to better health and reduced risk of many diseases [69,70,71], including cancer [72,73,74] and respiratory tract infections [75], as well as to better pregnancy and birth outcomes, such as preterm birth [76]. Also, the secondary analyses in two recently reported large clinical trials reported significantly reduced adverse outcomes: reduced incidence of cancer for people with body mass index <25 kg/m^2^ of body surface area taking 2000 IU/d of vitamin D_3_ [74] and reduced conversion from prediabetes to diabetes mellitus for people with body mass index <30 kg/m^2^ taking 4000 IU/d of vitamin D [77].

Limitations of our study include that the assays for the 25(OH)D concentration values used for the 15-country study varied between countries. Also, the standard deviations of the 25(OH)D concentrations were large for both data sets. The dietary supply data are approximations of the food actually consumed by people whose 25(OH)D concentrations were measured. Moreover, although food waste is generally estimated to be 30% of the supply [20], the rate of food wastage appears to be much higher in Saudi Arabia, where buffets are commonly served [78]. One report estimated food waste there to reach 50%, where annual food wastage per person is 427 kg in Saudi Arabia compared with 277 kg in the United States [79]. Finally, the external validity of the ecological study of postmenopausal women in Europe could be questioned. However, the results of this ecological study could lay a foundation for more careful observational studies.

## 5. Conclusions

Previous research studies have concluded that 25(OH)D concentrations are relatively low in the MENA region, as well as in Southern Europe. The risk factors that contribute to that public health burden are numerous and include the concealing dress code, owing to cultural and religious practices, as well as avoidance of direct sun exposure because of high temperatures in summer. However, the role of diet in developing profound hypovitaminosis D has not been adequately explored in these countries. Our study shows that dietary habits could play a minor role in the prevalence of hypovitaminosis D in the Middle East region. Serum 25(OH)D concentrations could be raised through a combination of vitamin D fortification of common food groups, such as dairy and grain products, as well as by advocating for vitamin D supplementation at the population level.

## Figures and Tables

**Table 1:** 25-hydroxyvitamin D (25(OH)D) concentration data used in this study for the set of 15 countries from the Middle East and Europe.

Country (City), Latitude	Age (years)	*N*	Year	Assay	25(OH)D (nmol/L), Summer *	25(OH)D (nmol/L), Winter *	Ref.
Bahrain, 43°	Mean 35	250 M 250 F	2010 2011	ELISA	27 ± 15 M 18 ± 15 F	41 ± 32 M 26 ± 24 F	[9]
Germany, 47°–49°	18–79	694 M su 748 M wi 770 F su 841 F wi	2008 2011	LIAISON, Roche	62 (59–65) M 59 (56–62) F	31 (29–34) M 35 (33–38) F	[26]
Iran (Babol), 36.5°	M 20–80 F 30–50	120 M 576 F	Sep 2010–Sep 2012	ELISA, lyophilized competitive protein binding	59 ± 29 M 52 ± 56 F	46 ± 58 M 48 ± 52 F	[27]
Iran (W. Azerbaijan), 37.5°	5–60	273 M 268 F	2015	ELISA, confirmed with HPLC	62 ± 5 M 50 ± 7 F	32 ± 4 M 30 ± 4 F	[28]
Israel, 31°	All ages	295,556 Jews	2009	LIAISON, DiaSorin	64 ± 24 M 50 ± 24 F	43 ± 24 M 41 ± 24 F	[29]
	All ages	59,203 Arabs	2009	LIAISON, DiaSorin	57 ± 22 M 38 ± 20 F	36 ± 22 M 24 ± 24 F	
Italy, 41.4°	49–74	1820 sum 1885 win 78% F	2014	LIAISON, DiaSorin	55 ± 21 M 72 ± 21 F	50 ± 20 M 50 ± 20 F	[30]
Jordan, 30.6°	18–45	23 M	?	Radioimmune, DiaSorin	44 ± 5 M	35 ± 4 M	[31]
	18–45	124 F		Radioimmune, DiaSorin	29 ± 5 F	25 ± 4 F	
Lebanon, 33.8°	30–50	74 M 318 F	2009–2010	Chemiluminescent, DiaSorin	51 ± 20 M + F	34 ± 20 M + F	[32]
Portugal (Porto), 41.2°	18–67	103 M 95 F	Jul/Aug 2015 Apr 2016	Elecsys, Cobas, Roche	70 ± 22 M 66 ± 22 F	43 ± 16 M 41 ± 16 F	[33]
Qatar, 23.4°	18–80	503 M 702 F	Dec 2012 Feb 2014	LIAISON, DiaSorin	42 ± 10 M + F	43 ± 10 M + F	[34]
Romania, 45.9°	Mean 50	1429 M 6569 F	2012–2016	Liaison XL, DiaSorin	66 ± 19 M 59 ± 16 F	38 ± 14 M 33 ± 13 F	[35]
Saudi Arabia, 23.9°	19+	659 F	2009	HPLC, Chromsystems, Germany	37 ± 2 F (±SE)		[36]
	19+	897 F	2009	HPLC, Chromsystems, Germany		34 ± 1 F (±SE)	
Sweden (Gothenburg), 57.7°	28–54	325 M 215 F	Oct 2009 Sep 2010	?	81 ± 27 M + F	48 ± 20 M + F	[37]
Switzerland, 47.1°	8–92	300 M 476 F	Sep 2011 Mar 2012	Immuno-diagnosticsystems, IDS	54 ± 20 M + F	42 ± 19 M + F	[38]
Syria, 34.8°	18–62	184 M 188 F	Apr 2011 Mar 2013	Elecsys 2010, Roche	37 ± 19 M 20 ± 14 F	23 ± 15 M 18 ± 9–23 F	[39]
Turkey (Ankara), 39.0°	21–52	53 M 65 F	Aug 2008 & Feb 2009	HPLC, AB Sciex, Foster City, CA, USA	72 ± 20 M 70 ± 30 F	38 ± 16 M 34 ± 17 F	[40]

*, mean, standard deviation; ELISA, enzyme-linked immunosorbent assay; F, female; HPLC, high-performance liquid chromatography; M, male; SE, standard error; su, summer; wi, winter; for Germany, numbers in parentheses are 95% confidence intervals.

**Table 2:** Dietary supply data for the 12 Middle East and European countries with data available from the Food and Agriculture Organization (FAO).

Country (City)	Latitude (°N)	Energy (kcal/Capita/Day) from:				
		Animal Fat	Eggs	Ocean Fish	Meat	Milk
Saudi Arabia	23.9	60	17	11	217	131
Jordan	30.6	23	29	7	140	115
Israel	31.0	16	37	20	418	250
Lebanon	33.8	16	22	17	242	160
Iran	36.5	20	21	10	143	74
	37.5	50	30	10	133	66
Turkey	39.0	15	33	8	138	309
Portugal	41.2	187	34	63	396	252
Italy	41.4	84	52	42	374	272
Romania	45.9	68	50	8	217	431
Switzerland	47.1	78	40	22	556	400
Germany	50.5	153	48	30	365	337
Sweden (Gothenburg)	57.7	51	49	43	357	402
Sweden (Stockholm)	60.0	47	43	47	344	428

**Table 3:** Data on dietary energy supply for animal products in 2002 for 19 European countries from the women with osteoporosis study [22].

Country	Energy (kcal/Capita/Day) from:				
	Animal Fat	Eggs	Ocean Fish	Meat	Milk
Greece	70	36	23	323	379
Spain	41	59	63	502	248
Italy	90	46	31	409	291
Bulgaria	51	42	8	234	239
Croatia	66	37	23	177	271
Romania	67	52	6	254	393
Hungary	285	66	5	419	214
France	85	60	56	559	393
Slovakia	210	47	12	245	153
Germany	147	48	32	355	249
Belgium	250	46	34	309	394
Poland	117	46	24	369	250
Netherlands	92	74	39	440	433
Lithuania	86	50	77	269	279
Denmark	199	66	48	381	276
Russian Fed.	37	52	29	240	239
Estonia	13	43	9	295	426
Norway	126	38	82	371	351
Finland	24	34	43	495	434

**Table 4:** Regression results for 25(OH)D for the set of 15 countries from the Middle East and Europe with respect to latitude, UVB dose, and dietary factors (Aug. 25).

Factor *	25(OH)D, Males (*R*, β, *p*)	25(OH)D, Females (R, β, *p*)
**Summer**		
Latitude	0.61, 0.80, 0.03	0.84, 1.45, <0.001
UVB	0.56, −3.0, 0.06	0.76, −5.2, 0.002
Eggs, milk	0.57, 0.044, 0.053	0.69, 0.087, 0.007
Milk	0.57, 0.047, 0.053	0.68, 0.093, 0.007
Eggs	0.50, 0.46, 0.12	0.60, 0.93, 0.02
Ocean fish	0.42, 0.25, 0.17	0.67, 0.67, 0.009
Meat	NS	0.33, 0.045, 0.25
Animal fat	NS	NS
**Winter**		
Latitude	0.36, 0.28, 0.25	0.62, 0.68, 0.02
UVB	0.51, −1.7, 0.09	0.37, −1.6, 0.20
Eggs, milk	0.55, 0.026, 0.07	NS
Milk	0.54, 0.027, 0.07	NS
Eggs	0.60, 0.36, 0.04	NS
Ocean fish	0.64, 0.23, 0.03	0.54, 0.34, 0.047
Meat	0.59, 0.027, 0. 04	NS
Animal fat	NS	NS

*, only 12 counties have dietary supply data; NS, not significant: adjusted r^2^ < 0.01; β, slope.

**Table 5:** Regression results for 20(OH)D concentration for 19 European countries from the European postmenopausal women’s study [22] with respect to dietary factors separately or in combination, latitude, and UVB dose.

Factor	25(OH)D Summer (*R*, β, *p*)	25(OH)D Winter * (*R*, β, *p*)
Animal fat, ocean fish, meat	0.74, 0.032, <0.001	0.46, 0.027, 0.09
Animal fat, ocean fish, eggs, meat	0.73, 0.030, <0.001	0.44, 0.025, 0.10
Animal fat, ocean fish	0.65, 0.049, 0.003	0.48, 0.05, 0.07
Animal fat, eggs, ocean fish	0.65, 0.046, 0.03	0.46, 0.048, 0.08
Ocean fish	0.54, 0.14, 0.02	0.63, 0.21, 0.01
Animal fat	0.51, 0.039, 0.03	0.32, 0.040, 0.25
Latitude	0.51, 0.45, 0.02	0.78, 0.90, <0.001
Meat	0.50, 0.030, 0.03	NS
UVB dose	0.47, −2.7, 0.04	0.56, −3.9, 0.03

*, data available for only 15 countries; NS, not significant: adjusted r^2^ < 0.01; β, slope.

**Table 6:** Cross-correlation for various factors with latitude for the 15-country study.

Factor	Latitude	Latitude (Omit Portugal)
Milk, eggs	0.80, 0.058, <0.001	0.80, 0.058, 0.001
UVB	0.89, −3.6, <0.001	0.89, −3.6, <0.001
Milk	0.79, 0.062, <0.001	0.79, 0.062, 0.001
Eggs	0.75, 0.67, 0.002	0.75, 0.67, 0.003
Ocean fish	0.57, 0.33, 0.03	0.73, 0.53, 0.005
Meat	0.43, 0.034, 0.12	0.44, 0.20, 0.13
Animal fat	0.30, 0.093, 0.29	0.42, 0.12, 0.15

**Table 7:** Cross-correlation for various factors with latitude for the 19-country study.

Factor	Latitude
UVB	0.78, −5.2, <0.001
Milk	0.47, 0.023, 0.27
Ocean fish	0.32, 0.098, 0.18
Eggs	NS
Meat	NS
Animal fat	NS

NS, not significant: adjusted r^2^ < 0.01.

**Table 8:** Influence of clothing style on 25(OH)D concentrations in the Middle East.

Country	Assay	Mean Age (years)	Veiled (nmol/L)	Not Veiled (nmol/L)	Western (nmol/L)	Ref.
Bahrain	ELISA	33	20 ± 19 Abayah		23 ± 19	[9]
Egypt *	IEMA radioimmune, DiaSorin		42 ± 8	58 ± 23		[45]
Jordan	Immunodiagnostic	39	29 ± 4 Niqab (7.3%) *			[46]
		34		31 ± 6 Hijab (46.7%) *		
		23			40 ± 8 (13.3%) *	
Lebanon	Radioimmune, Instar	39	13 ± 9 (47%) *	25 ± 16 (53%) *		[7]
	Protein-binding, DiaSorin	55	40 ± 19 (17%) *	68 ± 35 (83%) *		[47]
Syria	Elecsys 2010, Roche	36	13 (10–22 IQR) (60%) *	17 (10–33 IQR) (40%) *		[39]
Tunisia	Radioimmune, Instar	40?	35 ± ? (38%) *	43 ± ? (62%) *		[5]
UAE	chemiluminescent microparticle immunoassay, Abbott	45	44 ± 14 (38%) *	40 ± 13 (41%) *	47 ± 16 (21%)	[8]

*, percentage of participants in the study; ELISA, enzyme-linked immunosorbent assay; IQR, interquartile range.

## References

[1] Bassil D., Rahme M., Hoteit M., Fuleihan Gel H. (2013). Hypovitaminosis D in the Middle East and North Africa: Prevalence, risk factors and impact on outcomes. Derm. Endocrinol..

[2] Chakhtoura M., Rahme M., Chamoun N., El-Hajj Fuleihan G. (2018). Vitamin D in the Middle East and North Africa. Bone Rep..

[3] Lips P., Cashman K.D., Lamberg-Allardt C., Bischoff-Ferrari H.A., Obermayer-Pietsch B.R., Bianchi M., Stepan J., El-Hajj Fuleihan G., Bouillon R. (2019). Management of endocrine disease: Current vitamin D status in European and Middle East countries and strategies to prevent vitamin D deficiency; a position statement of the European Calcified Tissue Society. Eur. J. Endocrinol..

[4] Grant W.B., Bhattoa H.P., Pludowski P., Feldman D. (2018). Determinants of Vitamin D Deficiency From Sun Exposure: A Global Perspective. Vitamin D.

[5] Meddeb N., Sahli H., Chahed M., Abdelmoula J., Feki M., Salah H., Frini S., Kaabachi N., Belkahia C., Mbazaa R. (2005). Vitamin D deficiency in Tunisia. Osteoporos. Int..

[6] Batieha A., Khader Y., Jaddou H., Hyassat D., Batieha Z., Khateeb M., Belbisi A., Ajlouni K. (2011). Vitamin D status in Jordan: Dress style and gender discrepancies. Ann. Nutr. Metab..

[7] Gannage-Yared M.H., Chemali R., Yaacoub N., Halaby G. (2000). Hypovitaminosis D in a sunny country: Relation to lifestyle and bone markers. J. Bone Miner. Res..

[8] Al Attia H.M., Ibrahim M.A. (2012). The high prevalence of vitamin D inadequacy and dress style of women in the sunny UAE. Arch. Osteoporos..

[9] Golbahar J., Al-Saffar N., Altayab Diab D., Al-Othman S., Darwish A., Al-Kafaji G. (2014). Predictors of vitamin D deficiency and insufficiency in adult Bahrainis: A cross-sectional study. Public Health Nutr..

[10] Ardawi M.S., Sibiany A.M., Bakhsh T.M., Qari M.H., Maimani A.A. (2012). High prevalence of vitamin D deficiency among healthy Saudi Arabian men: Relationship to bone mineral density, parathyroid hormone, bone turnover markers, and lifestyle factors. Osteoporos. Int..

[11] Hwalla N., Al Dhaheri A.S., Radwan H., Alfawaz H.A., Fouda M.A., Al-Daghri N.M., Zaghloul S., Blumberg J.B. (2017). The Prevalence of Micronutrient Deficiencies and Inadequacies in the Middle East and Approaches to Interventions. Nutrients.

[12] Khosravi-Boroujeni H., Sarrafzadegan N., Sadeghi M., Roohafza H., Ng S.K., Pourmogaddas A., Ahmed F. (2017). Prevalence and Trends of Vitamin D Deficiency among Iranian Adults: A Longitudinal Study from 2001–2013. J. Nutr. Sci. Vitaminol..

[13] Pilz S., Marz W., Cashman K.D., Kiely M.E., Whiting S.J., Holick M.F., Grant W.B., Pludowski P., Hiligsmann M., Trummer C. (2018). Rationale and Plan for Vitamin D Food Fortification: A Review and Guidance Paper. Front. Endocrinol..

[14] Gannage-Yared M.H., Chemali R., Sfeir C., Maalouf G., Halaby G. (2005). Dietary calcium and vitamin D intake in an adult Middle Eastern population: Food sources and relation to lifestyle and PTH. Int. J. Vitam. Nutr. Res..

[15] Crowe F.L., Steur M., Allen N.E., Appleby P.N., Travis R.C., Key T.J. (2011). Plasma concentrations of 25-hydroxyvitamin D in meat eaters, fish eaters, vegetarians and vegans: Results from the EPIC-Oxford study. Public Health Nutr..

[16] Liu J., Greenfield H., Strobel N., Fraser D.R. (2013). The influence of latitude on the concentration of vitamin D3 and 25-hydroxy-vitamin D3 in Australian red meat. Food Chem..

[17] Taylor C.L., Patterson K.Y., Roseland J.M., Wise S.A., Merkel J.M., Pehrsson P.R., Yetley E.A. (2014). Including food 25-hydroxyvitamin D in intake estimates may reduce the discrepancy between dietary and serum measures of vitamin D status. J. Nutr..

[18] Dunlop E., Cunningham J., Sherriff J.L., Lucas R.M., Greenfield H., Arcot J., Strobel N., Black L.J. (2017). Vitamin D(3) and 25-Hydroxyvitamin D(3) Content of Retail White Fish and Eggs in Australia. Nutrients.

[19] Food and Agriculture Organization of the United Nations Food Balance Sheets. http://www.fao.org/faostat/en/#data/FBS.

[20] FAO (2011). Global Food Losses and Food Waste—Extent, Causes and Prevention.

[21] Armstrong B., Doll R. (1975). Environmental factors and cancer incidence and mortality in different countries, with special reference to dietary practices. Int. J. Cancer.

[22] Kuchuk N.O., van Schoor N.M., Pluijm S.M., Chines A., Lips P. (2009). Vitamin D status, parathyroid function, bone turnover, and BMD in postmenopausal women with osteoporosis: Global perspective. J. Bone Miner. Res..

[23] Dafterdar R., Al-Fayoumi M., Saadeddin S., Khan R., Alothaim A., Hasanato R., Al-Shangiti A., Fakhoury H., Tamimi W. (2014). Vitamin D immunoassay systems: A comparison. Br. J. Biomed. Sci..

[24] Sadat-Ali M., Al-Elq A.H., Al-Shaikh I.H., Al-Turki H.A., Al-Ali A.K., Al-Othman A.A. (2014). Assessment of low vitamin D among Saudi Arabians. Did we overshoot the runway?. Saudi Med. J..

[25] Garland C.F., Cuomo R.E., Gorham E.D., Zeng K., Mohr S.B. (2016). Cloud cover-adjusted ultraviolet B irradiance and pancreatic cancer incidence in 172 countries. J. Steroid Biochem. Mol. Biol..

[26] Rabenberg M., Scheidt-Nave C., Busch M.A., Rieckmann N., Hintzpeter B., Mensink G.B. (2015). Vitamin D status among adults in Germany--results from the German Health Interview and Examination Survey for Adults (DEGS1). BMC Public Health.

[27] Heidari B., Mirghassemi M.B.H. (2012). Seasonal variations inserum vitamin D according to age and sex. Casp. J. Interrnal Med..

[28] Nouri Saeidlou S., Vahabzadeh D., Babaei F., Vahabzadeh Z. (2017). Seasonal variations of vitamin D and its relation to lipid profile in Iranian children and adults. J. Health Popul. Nutr..

[29] Saliba W., Rennert H.S., Kershenbaum A., Rennert G. (2012). Serum 25(OH)D concentrations in sunny Israel. Osteoporos. Int..

[30] Bonelli P., Buonocore R., Aloe R., Lippi G. (2016). Blood Sampling Seasonality as an Important Preanalytical Factor for Assessment of Vitamin D Status. J. Med. Biochem..

[31] Mishal A.A. (2001). Effects of different dress styles on vitamin D levels in healthy young Jordanian women. Osteoporos. Int..

[32] Gannage-Yared M.H., Helou E., Zaraket V., Abi Akl S., Antonios L., Moussalli M.L., Wakim S. (2014). Serum 25 hydroxyvitamin D in employees of a Middle Eastern university hospital. J. Endocrinol. Investig..

[33] Bettencourt A., Boleixa D., Reis J., Oliveira J.C., Mendonca D., Costa P.P., Silva B.M.D., Marinho A., Silva A.M.D. (2018). Serum 25-hydroxyvitamin D levels in a healthy population from the North of Portugal. J. Steroid Biochem. Mol. Biol..

[34] Al-Dabhani K., Tsilidis K.K., Murphy N., Ward H.A., Elliott P., Riboli E., Gunter M., Tzoulaki I. (2017). Prevalence of vitamin D deficiency and association with metabolic syndrome in a Qatari population. Nutr. Diabetes.

[35] Niculescu D.A., Capatina C.A.M., Dusceac R., Caragheorgheopol A., Ghemigian A., Poiana C. (2017). Seasonal variation of serum vitamin D levels in Romania. Arch. Osteoporos..

[36] Kanan R.M., Al Saleh Y.M., Fakhoury H.M., Adham M., Aljaser S., Tamimi W. (2013). Year-round vitamin D deficiency among Saudi female out-patients. Public Health Nutr..

[37] Klingberg E., Olerod G., Konar J., Petzold M., Hammarsten O. (2015). Seasonal variations in serum 25-hydroxy vitamin D levels in a Swedish cohort. Endocrine.

[38] Merlo C., Trummler M., Essig S., Zeller A. (2015). Vitamin D Deficiency in Unselected Patients from Swiss Primary Care: A Cross-Sectional Study in Two Seasons. PLoS ONE.

[39] Sayed-Hassan R., Abazid N., Alourfi Z. (2014). Relationship between 25-hydroxyvitamin D concentrations, serum calcium, and parathyroid hormone in apparently healthy Syrian people. Arch. Osteoporos..

[40] Cinar N., Harmanci A., Yildiz B.O., Bayraktar M. (2014). Vitamin D status and seasonal changes in plasma concentrations of 25-hydroxyvitamin D in office workers in Ankara, Turkey. Eur. J. Intern. Med..

[41] Jablonski N.G., Chaplin G. (2000). The evolution of human skin coloration. J. Hum. Evol..

[42] Chaplin G. (2004). Geographic distribution of environmental factors influencing human skin coloration. Am. J. Phys. Anthropol..

[43] Chaplin G., Jablonski N.G. (2013). The human environment and the vitamin D compromise: Scotland as a case study in human biocultural adaptation and disease susceptibility. Hum. Biol..

[44] Jablonski N.G., Chaplin G. (2010). Colloquium paper: Human skin pigmentation as an adaptation to UV radiation. Proc. Natl. Acad. Sci. USA.

[45] Botros R.M., Sabry I.M., Abdelbaky R.S., Eid Y.M., Nasr M.S., Hendawy L.M. (2015). Vitamin D deficiency among healthy Egyptian females. Endocrinol. Nutr..

[46] Mallah E.M., Hamad M.F., Elmanaseer M.A., Qinna N.A., Idkaidek N.M., Arafat T.A., Matalka K.Z. (2011). Plasma concentrations of 25-hydroxyvitamin D among Jordanians: Effect of biological and habitual factors on vitamin D status. BMC Clin. Pathol..

[47] Darwish H., Zeinoun P., Ghusn H., Khoury B., Tamim H., Khoury S.J. (2015). Serum 25-hydroxyvitamin D predicts cognitive performance in adults. Neuropsychiatr. Dis. Treat..

[48] Grant W.B. (2014). A multicountry ecological study of cancer incidence rates in 2008 with respect to various risk-modifying factors. Nutrients.

[49] Grant W.B. (1997). Dietary links to Alzheimer’s disease. Alz. Dis. Rev..

[50] Grant W.B. (1998). The APOE-epsilon4 allele and Alzheimer disease among African Americans, Hispanics, and whites. JAMA J. Am. Med. Assoc..

[51] Luchsinger J.A., Tang M.X., Shea S., Mayeux R. (2002). Caloric intake and the risk of Alzheimer disease. Arch. Neurol..

[52] Rainey-Smith S.R., Gu Y., Gardener S.L., Doecke J.D., Villemagne V.L., Brown B.M., Taddei K., Laws S.M., Sohrabi H.R., Weinborn M. (2018). Mediterranean diet adherence and rate of cerebral Abeta-amyloid accumulation: Data from the Australian Imaging, Biomarkers and Lifestyle Study of Ageing. Transl. Psychiatry.

[53] Morris M.C., Evans D.A., Bienias J.L., Tangney C.C., Bennett D.A., Wilson R.S., Aggarwal N., Schneider J. (2003). Consumption of fish and n-3 fatty acids and risk of incident Alzheimer disease. Arch. Neurol..

[54] Grant W.B. (2016). Using Multicountry Ecological and Observational Studies to Determine Dietary Risk Factors for Alzheimer’s Disease. J. Am. Coll. Nutr..

[55] Grant W.B. (1998). Reassessing the role of sugar in the etiology of heart disease. J. Orthomolec. Med..

[56] Johnson R.K., Appel L.J., Brands M., Howard B.V., Lefevre M., Lustig R.H., Sacks F., Steffen L.M., Wylie-Rosett J. (2009). Dietary sugars intake and cardiovascular health: A scientific statement from the American Heart Association. Circulation.

[57] Moukayed M., Grant W.B. (2013). Molecular link between vitamin D and cancer prevention. Nutrients.

[58] Jakobsen J., Smith C., Bysted A., Cashman K.D. (2019). Vitamin D in Wild and Farmed Atlantic Salmon (Salmo Salar)-What Do We Know?. Nutrients.

[59] Chuang S.C., Chen H.L., Tseng W.T., Wu I.C., Hsu C.C., Chang H.Y., Chen Y.I., Lee M.M., Liu K., Hsiung C.A. (2016). Circulating 25-hydroxyvitamin D and physical performance in older adults: A nationwide study in Taiwan. Am. J. Clin. Nutr..

[60] Lehmann U., Gjessing H.R., Hirche F., Mueller-Belecke A., Gudbrandsen O.A., Ueland P.M., Mellgren G., Lauritzen L., Lindqvist H., Hansen A.L. (2015). Efficacy of fish intake on vitamin D status: A meta-analysis of randomized controlled trials. Am. J. Clin. Nutr..

[61] Farrell C.J., Martin S., McWhinney B., Straub I., Williams P., Herrmann M. (2012). State-of-the-art vitamin D assays: A comparison of automated immunoassays with liquid chromatography-tandem mass spectrometry methods. Clin. Chem..

[62] Holmes E.W., Garbincius J., McKenna K.M. (2013). Analytical variability among methods for the measurement of 25-hydroxyvitamin D: Still adding to the noise. Am. J. Clin. Pathol..

[63] Hsu S.A., Soldo J., Gupta M. (2013). Evaluation of two automated immunoassays for 25-OH vitamin D: Comparison against LC-MS/MS. J. Steroid Biochem. Mol. Biol..

[64] Wyness S.P., Straseski J.A. (2015). Performance characteristics of six automated 25-hydroxyvitamin D assays: Mind your 3s and 2s. Clin. Biochem..

[65] Cashman K.D., Dowling K.G., Skrabakova Z., Kiely M., Lamberg-Allardt C., Durazo-Arvizu R.A., Sempos C.T., Koskinen S., Lundqvist A., Sundvall J. (2015). Standardizing serum 25-hydroxyvitamin D data from four Nordic population samples using the Vitamin D Standardization Program protocols: Shedding new light on vitamin D status in Nordic individuals. Scand. J. Clin. Lab. Investig..

[66] Sempos C.T., Durazo-Arvizu R.A., Binkley N., Jones J., Merkel J.M., Carter G.D. (2016). Developing vitamin D dietary guidelines and the lack of 25-hydroxyvitamin D assay standardization: The ever-present past. J. Steroid Biochem. Mol. Biol..

[67] Sarafin K., Durazo-Arvizu R., Tian L., Phinney K.W., Tai S., Camara J.E., Merkel J., Green E., Sempos C.T., Brooks S.P. (2015). Standardizing 25-hydroxyvitamin D values from the Canadian Health Measures Survey. Am. J. Clin. Nutr..

[68] Pludowski P., Holick M.F., Grant W.B., Konstantynowicz J., Mascarenhas M.R., Haq A., Povoroznyuk V., Balatska N., Barbosa A.P., Karonova T. (2018). Vitamin D supplementation guidelines. J. Steroid Biochem. Mol. Biol..

[69] Holick M.F. (2007). Vitamin D deficiency. N. Engl. J. Med..

[70] Pludowski P., Holick M.F., Pilz S., Wagner C.L., Hollis B.W., Grant W.B., Shoenfeld Y., Lerchbaum E., Llewellyn D.J., Kienreich K. (2013). Vitamin D effects on musculoskeletal health, immunity, autoimmunity, cardiovascular disease, cancer, fertility, pregnancy, dementia and mortality—A review of recent evidence. Autoimmun. Rev..

[71] Grant W.B. (2019). Vitamin D and health in the Mediterranean countries. Hormones.

[72] McDonnell S.L., Baggerly C., French C.B., Baggerly L.L., Garland C.F., Gorham E.D., Lappe J.M., Heaney R.P. (2016). Serum 25-Hydroxyvitamin D Concentrations >/=40 ng/mL Are Associated with >65% Lower Cancer Risk: Pooled Analysis of Randomized Trial and Prospective Cohort Study. PLoS ONE.

[73] McDonnell S.L., Baggerly C.A., French C.B., Baggerly L.L., Garland C.F., Gorham E.D., Hollis B.W., Trump D.L., Lappe J.M. (2018). Breast cancer risk markedly lower with serum 25-hydroxyvitamin D concentrations >/=60 vs <20 ng/mL (150 vs 50 nmol/L): Pooled analysis of two randomized trials and a prospective cohort. PLoS ONE.

[74] Manson J.E., Cook N.R., Lee I.M., Christen W., Bassuk S.S., Mora S., Gibson H., Gordon D., Copeland T., D’Agostino D. (2019). Vitamin D Supplements and Prevention of Cancer and Cardiovascular Disease. N. Engl. J. Med..

[75] Martineau A.R., Jolliffe D.A., Hooper R.L., Greenberg L., Aloia J.F., Bergman P., Dubnov-Raz G., Esposito S., Ganmaa D., Ginde A.A. (2017). Vitamin D supplementation to prevent acute respiratory tract infections: Systematic review and meta-analysis of individual participant data. BMJ.

[76] McDonnell S.L., Baggerly K.A., Baggerly C.A., Aliano J.L., French C.B., Baggerly L.L., Ebeling M.D., Rittenberg C.S., Goodier C.G., Mateus Nino J.F. (2017). Maternal 25(OH)D concentrations >/=40 ng/mL associated with 60% lower preterm birth risk among general obstetrical patients at an urban medical center. PLoS ONE.

[77] Pittas A.G., Dawson-Hughes B., Sheehan P., Ware J.H., Knowler W.C., Aroda V.R., Brodsky I., Ceglia L., Chadha C., Chatterjee R. (2019). Vitamin D Supplementation and Prevention of Type 2 Diabetes. N. Engl. J. Med..

[78] Baig M.B., Al-Zahrani K.H., Schneider F., Straquadine G.S., Mourad M. (2018). Food waste posing a serious threat to sustainability in the Kingdom of Saudi Arabia—A systematic review. Saudi J. Biol. Sci..

[79] FAO (2014). Reducing Food Losses and Waste in the Near East & North Africa Region.

